# Research on the Isolation of Endophytic Fungi from Papaya and the Prevention of *Colletotrichum gloeosporioides*

**DOI:** 10.3390/jof10080550

**Published:** 2024-08-05

**Authors:** Jinhui Lv, Shuwei Ke, Xinrui He, Baolong Zhang, Zhongbing Zheng, Ping Chen

**Affiliations:** 1 School of Breeding and Multiplication (Sanya Institute of Breeding and Multiplication), Hainan University, Sanya 572025, China; lvjinhuixue@163.com (J.L.); zhbl2248@hotmail.com (B.Z.); 2 Key Laboratory for Quality Regulation of Tropical Horticultural Crops of Hainan Province, School of Tropical Agriculture and Forestry, Hainan University, Haikou 570228, China; keshuwei4901@163.com (S.K.); 18996086216@139.com (X.H.)

**Keywords:** *Carica papaya* L., endophytic fungi, antioxidant activity, *Colletotrichum gloeosporioides*, defense enzyme

## Abstract

Endophytic fungi can be used as a source of herbal antioxidants to overcome the limitations of low yield and lengthy growth cycles associated with using plants as raw materials for antioxidant production. Papaya fruit is often susceptible to infection by *Colletotrichum gloeosporioides* after harvest, leading to postharvest rot. Endophytic fungi were extracted with ethyl acetate, and the initial screening concentration was 100 mg/L. Seven strains were identified, with scavenging rates exceeding 50% and strong antioxidant activity. The IC50 values in DPPH and ABTS free radical scavenging assays ranged from 19.72 to 84.06 mg/L and from 14.34 to 64.63 mg/L, respectively. Strain Y17 exhibited robust antioxidant activity (IC50 < 20 mg/L) and was identified as *Penicillium rolfsii* (MT729953) through ITS sequencing. Treatment of papaya fruit wounds with a fermentation broth of strain Y17 significantly inhibited the infection and colonization of anthracnose pathogens, resulting in a slowed disease incidence rate. This promoted the activity of protective enzymes, such as CAT, POD, and SOD, in the papaya fruit and slowed down the rate of MDA accumulation. This strain, which was found to have antioxidant activity in this study, has the potential to control anthracnose in papaya and has value in terms of further development and utilization.

## 1. Introduction

Endophytic fungi, a class of fungi residing within plants throughout all or part of their life cycle, exhibit a unique trait, namely they do not induce noticeable disease symptoms in host plant tissues [1]. Representing a novel microbial resource, endophytic fungi have the ability to generate metabolites with distinctive structures and components, due to their prolonged survival within the specialized microenvironmental structures of host plants [2,3]. The extraction of biologically active compounds from plant endophytic fungi opens up novel avenues to address challenges associated with extended plant growth periods and low active component yields. This presents significant practical applications and developmental potential [4,5]. Tan et al. isolated more than 376 genera of plant endophytic fungi from various tissue components of 212 medicinal plants alone, encompassing major fungal types such as *Basidiomycetes* and *Ascomycetes* [4]. In a study by Li et al., 27 endophytic fungi were isolated from distinct parts of the roots, stems, and leaves of *Bupleurum chinense*, representing nine different genera [6]. Chen et al. conducted a comparative analysis of the diversity of endophytic fungi in licorice (*Glycyrrhiza uralensis*) originating from five different locations: Beijing, Gansu, Ningxia, Inner Mongolia, and Xinjiang. They observed variations in the abundance and taxonomic composition of endophytic fungi in licorice from different geographical origins [7]. It can be inferred that the prevalence and diversity of plant endophytic fungi contribute significantly to the wealth of microbial resources available for scientific investigation. Moreover, the extraction of potent bioactive compounds from the secondary metabolites of endophytic fungi introduces a novel approach to overcoming the challenges associated with the prolonged growth cycle of plant resources [8,9], the meager yield of active components, and the depletion of non-renewable resources. This holds significant application value and developmental potential [10,11]. Throughout the extensive evolutionary process, a mutually beneficial symbiotic relationship gradually developed between endophytic fungi and the host plant. Endophytic fungi and their metabolites serve as a newfound reservoir for screening and exploiting antimicrobial strains. For instance, Ge et al. isolated a high-antifungal-activity strain of *Penicillium oxalicum* from Ginkgo (*Ginkgo biloba* L.) and separated trichothecene, which displays broad-spectrum antifungal activity, through meticulous techniques, such as column chromatography, thin-layer chromatography, and preparative high-performance liquid chromatography [12]. Wang et al. observed a profound inhibitory effect on *Staphylococcus aureus* in the fermentation broth of an endophytic fungus, *Penicillium turbatum*, which was isolated from *Macleaya cordata (Willd.) R*. Br. They optimized the plate assay test for the fermentation broth, revealing an inhibition circle diameter of 52.36 ± 0.88 mm. This indicated a substantial inhibitory effect [13]. Similarly, Chen et al. studied the fermentation broth of the endophytic fungus VDL117. The maximum inhibition rate of the fermentation broth against five kinds of fungi (*Fusarium oxysporum sp. vasinfectum*, *F. semani*, *Alternaria brassicae*, *F. xysporium sp. cucumebrium*, and *Magnaporthe*) was 27.49%, 23.26%, 18.27%, 13.98%, and 13.04%, respectively [14]. Some endophytic fungi associated with plants not only induce a plant’s innate resistance, but also activate its autonomous defense mechanisms against adverse factors, bolstering its resilience to pests and diseases [15]. As a class of biocontrol organisms, endophytic fungi exhibit remarkable advantages for biological control within agricultural production. Consequently, the judicious utilization of endophytic fungi for biocontrol presents significant practical value in safeguarding authentic agricultural production activities [16].

*Carica papaya* L., a ubiquitous tropical and subtropical herbaceous fruit tree [17], stands out for its rich composition of amino acids, vitamin C, papain, carpaine, and other constituents. Beyond its role as a fresh fruit, papaya also holds medicinal significance [18,19]. Empirical studies have revealed the presence of alkaloids, papain, phenolic compounds, terpenoids, flavonoids [20], and other bioactive components [21] in papaya fruit and leaves. These components exhibit varying degrees of antifungal [22], antioxidant, anti-tumor, and immune-regulatory effects. However, despite its numerous attributes, papaya fruit is susceptible to infestation by various pathogenic microorganisms after harvest, leading to decay. Among these pathogens, *Colletotrichum gloeosporioides* emerges as the primary culprit, responsible for postharvest anthracnose in papaya [23]. While there has been extensive research on the application of endophytic microorganisms in papaya for plant disease control and growth promotion, these studies have predominantly focused on endophytic bacteria, with limited reports on endophytic fungi. Shi et al. identified a bacterial strain, *Pseudomonas putida* biovar I, for the organic management of endophytic microorganisms in postharvest disease applications for papaya. In a subsequent efficacy test, the fungus exhibited a remarkable level of effectiveness of 57.4% against anthracnose [24]. Furthermore, in 2011, an endophytic strain of *Pseudomonas aeruginosa* was identified, demonstrating a significant reduction in the incidence of anthracnose in efficacy tests [25].

This study focused on the isolation of endophytic fungi from various segments of the papaya plant through tissue separation, and both morphological and molecular biological methodologies were employed for identification. This research delved into the distribution of endophytic fungi across distinct parts of the papaya plant, the composition of different endophytic fungi, and their ecological distribution. Screening for antioxidant activity in the endophytic fungi of papaya was conducted by using DPPH and ABTS techniques, and determination of the total reduction capacity of an ethyl acetate extract was performed using the potassium ferricyanide method. The endophytic fungi that were isolated from papaya were subsequently utilized to confirm the biological control efficacy against papaya anthracnose through flat-panel tests and postharvest control experiments. This investigation lays the groundwork for the development and utilization of the resources of endophytic fungi from papaya.

## 2. Materials and Methods

### 2.1. Materials

#### 2.1.1. Plant Materials and Pathogens

The sampling sites were located in the Agricultural Science Base of Haidian Campus at Hainan University. Leaves, branches, and fruits of distinctive papaya plants were randomly collected. The samples were put into a sterile plastic bag and brought back to the laboratory for the isolation of endophytic fungi within 24 h.

Fruit selection: Healthy papaya fruits with no apparent disorders or spots, no apparent mechanical injuries, and extraordinarily constant measurements and maturity were selected. The papaya fruits were produced in Danzhou, Hainan Province, and the papaya variety was “RISHENG”.

The pathogens used in the tests were as follows: *Colletotrichum gloeosporioides* penz., *Pestalotiopsis* spp., *Fusarium oxysporum*, *Botryodiplodia theobromae*, and *Neoscytalidium dimidiatum*. These strains were obtained from the Chinese Academy of Tropical Agricultural Sciences, the Agricultural Culture Collection of China, and the laboratory’s own depository.

#### 2.1.2. Drugs and Chemicals

The methanol, ethanol, sodium hypochlorite, ethyl acetate, the above reagents were purchased from Xilong scientific (Guangzhou, China). 2,2-biazol-bis (3-ethyl-benzothiazole-6-sulfonic acid) diammonium salt (ABTS), 1, 1-diphenyl-2-azide (DPPH), 2, 6-di-tert-butyl-4-methylphenol (BHT), and water-soluble vitamin C (vitamin C), the above reagents were purchased from Solarbio (Beijing, China), were all pure and above-grade reagents for analysis. Goldview, DNAmarker (Vazyme, Nanjing, China), etc. were bought from Beijing Solarbio Technology Co., Ltd. (Beijing, China) for the identification of the molecular biology of the strains. Potato glucose agar (PDA) and potato glucose broth (PDB) media were bought from Beijing Solarbio Technology Co., Ltd. (Beijing, China). PDA was used for the isolation and purification of endophytic fungi. PDB was used for the liquid culture of endophytic fungi and the shaking-flask fermentation of the strains.

### 2.2. Methods

#### 2.2.1. Isolation, Purification, and Identification of Strains

The endophytic fungi in various parts of papaya were isolated using a conventional tissue separation method, in which the leaves, stems, and fruits were rinsed with running water [26]. Subsequently, on an ultra-clean working table, these samples were cut to size with a sterile blade. These blocks were then immersed in a 75% ethanol solution for 75 s, followed by a 2.6% sodium hypochlorite solution for 150 s. The samples underwent a series of 3–5 ddH_2_O rinses for surface disinfection. Afterward, the samples were further cut into 0.5 cm × 0.5 cm tissue blocks with a sterile blade. These were then transferred onto potato dextrose agar (PDA), containing chloramphenicol, for cultivation. Notably, 3 replicates were placed in each culture medium. Thermo-regulated cultivation at 25 °C was sustained for 5 to 7 days within a biochemical incubator, after which the bacteria were identified, documented, and recorded. The fungal growth status of the plant tissue blocks was observed at regular intervals; the mycelium was picked up with an inoculation needle once it grew out, and it was promptly transferred to a new PDA plate for purification. To ensure that the isolated strains were endophytic fungi from papaya, three control treatments were set up in this experiment. These were intended to test the effectiveness of the surface disinfection to ensure that the strains isolated from papaya were plant endophytic fungi. The specific procedures were as outlined below.

Environmental control: Three open PDA culture dishes were placed in a laminar-flow hood throughout the process of isolating the endophytic fungi, as environmental controls. If no microbial growth was observed after 7 days of incubation, it was proven that environmental microbial interference had been eliminated.

Liquid-rinsing test method: After strict disinfection procedures on the plant tissue blocks, they were rinsed with sterile water, and the final water used to rinse them was transferred to a PDA plate. This was incubated at 25 °C for 7 days; if no microbial growth was observed after 7 days, this indicated that the rinsing liquid was sterile and that the plant tissue was thoroughly disinfected.

Tissue imprinting method: The surface-sterilized tissue blocks were placed on a PDA plate, and the sterilized plant tissue blocks were allowed to contact the PDA culture medium for 5 min. This was followed by incubation at 25 °C for 7 days; if no microbial growth was observed after 7 days, this indicated that the sample surface was sterile and that the plant tissue blocks were thoroughly disinfected.

This experiment was repeated three times, with 9 random samples of papaya fruits, leaves, and stems being selected each time. Each sample was inoculated on 3 plates, with 3 tissue blocks placed on each plate, totaling 243 tissue blocks inoculated from each part, and 729 tissue blocks that were inoculated in total. Meanwhile, the isolation rate of the endophytic fungi in different parts of the papaya was recorded. The formula was as follows:

Isolation rate = (number of strains isolated from tissue blocks/total number of tissue blocks) × 100%

Purification of the endophytic fungi from papaya: When the mycelium grew around the tissue blocks of different parts of the papaya plants that were placed on PDA plates, it was promptly taken from around the tissue blocks with an inoculation needle and transferred to a new PDA plate for cultivation at 25 °C for 10 days. The purification steps mentioned above were repeated until pure fungal colonies were obtained; their color, size, texture, mycelium, and other morphological characteristics were observed and recorded, and they were assigned numbers.

The purified strains were inoculated on a bevel medium of PDA, which was sealed with liquid paraffin and stored in a refrigerator at 4 °C. The isolation rate (IR), signifying the ratio of the number of strains isolated from the samples to the total number of samples, was calculated by dividing the number of endophytic fungal strains isolated by the total number of tissues blocks in this experiment.

The morphological characteristics of the endophytic fungal strains were ascertained through insertion and staining with a solution of cotton blue. The morphological features of the conidiophores and conidia of the fungi were meticulously examined at magnifications of 400 and 1000, using a Leica DM2000 compound microscope (Wetzlar, Germany). Genomic DNA extracted from the endophytic fungi of papaya served as the template for PCR amplification. Amplification was achieved using ITS1F forward (5′-CTTGGTCATTTAGAGAAGTAA-3′) and ITS4 reverse (5′-TCCTCCGCTTAGATATGC-3′) primers. The purified PCR product underwent sequence determination by a specialized agency. The determined ITS sequences were subjected to a similarity comparison using the NCBI database, with the BLAST program. If the sequence similarity was greater than 99%, the same species was identified. If the sequence was between 95% and 99%, the samples were identified as the same genus. If the sequence was less than 95%, the samples were identified as the same family. The results of the molecular identification were obtained [27]. The relative frequency (RF), which denotes the ratio of positive endophytic fungal strains to the total number of isolated strains, was calculated as the percentage of positive strains among the entire isolated endophytic fungal population [28,29].

#### 2.2.2. Strain Fermentation and Sample Preparation

A single strain that grew well on a PDA plate was selected, and a sterile perforator with a diameter of about 6 mm was used to punch holes in the PDA plate on an aseptic operating table. Three to five mushroom cakes were inoculated into 250 mL of PDB broth after sterilization and cooling at 28 °C, and they were oscillated at 130 r/min for 10 days. After fermentation for 10 days, the fermentation liquid and mycelium were separated through vacuum filtration. The obtained fermentation liquid was soaked overnight with an equal-volume ethyl acetate solution, which was used for extraction 3 times. Then, the extraction liquid was collected, recovered, and concentrated to 1/10 of the original volume with a rotary evaporator at 50 °C. The obtained mycelium was dried at 55 °C and ground into a powder. The mass–volume ratio (*w*/*v*) of the mycelium and ethyl acetate was a solid–liquid ratio of 1:20. After ultrasonic treatment for 1 h, the insoluble matter was removed via filtration, recovered, and concentrated under pressure. An ethyl acetate extract of the strain was obtained by combining the fermentation solution and the concentrated extract of mycelium and evaporating until dry at 50 °C. It was stored in a refrigerator at 4 °C for future use.

#### 2.2.3. Screening of the Strains with Antioxidant Activity and Determination of the Half-Maximal Inhibitory Concentration (IC50)

The capacity of the secondary metabolites of each strain to scavenge ABTS free radicals was determined through the TEAC (Trolox equivalent antioxidant capacity) technique [30,31]. An ABTS solution of 7.4 mmol/L and a potassium persulfate solution of 2.6 mmol/L were prepared. The ABTS mother liquor was prepared by mixing the above two solutions in identical proportions and storing them in the dark for 12 to 16 h. The mother liquor was diluted with anhydrous alcohol to 0.7 ± 0.05 at 734 nm; then, the ABTS reaction solution was prepared. A 0.6 mL template and 2.4 mL of the ABTS reaction solution were mixed well. The absorbance was examined precisely after a reaction time of 6 min. A methanol solution was used as a blank control. All determinations were carried out in triplicate.

The potential to scavenge DPPH free radicals was determined based on the approach by Zhang et al., with moderate modifications [32,33]. For the preparation of a 15 mmol/L DPPH working solution (with dissolved methanol), 2 mL of DPPH solution and 1 mL of sample solution were combined in a test tube, and the absorbance was determined at a wavelength of 517 nm after 30 min of reaction. All determinations were carried out in triplicate. Strains with clearance rates greater than 50% at a sample concentration of 0.1 g/L in the ABTS and DPPH free radical scavenging tests were weighed using the weight loss method to prepare ethyl acetate extracts of each strain at concentrations of 100, 50, 25, 12.5, and 6.25 mg/L. As positive controls, vitamin C and the synthetic antioxidant BHT were prepared in methanol at concentrations of 100, 50, 25, 12.5, and 6.25 mg/L. The clearance rates were determined according to the same steps as those used for ABTS and DPPH free radicals; this experiment was repeated in triplicate. The IC50 value for each strain used in the test was calculated when the clearance rates for DPPH and ABTS reached 50%.

The complete reduction capacity of potassium ferricyanide was measured [34]. The ethyl acetate extract that was obtained by fermenting each strain was configured as a 1 mg/mL methanol solution. Then, 1 mL of the pre-configured sample solution was delivered to a test tube, and 1.0 mL of a pH 6.6 phosphate buffer and 1.0 mL of a 1% potassium ferricyanide solution were sequentially added at 50 °C for 20 min. After cooling with water, 1 mL of 10% trichloroacetic acid solution was added and mixed well. Then, 2.5 mL of supernatant was added to a new centrifuge tube, and 2.5 mL of methanol and 0.5 mL of 0.1% ferric chloride solution were added and mixed well. The absorption value was measured at 700 nm, after the solution was allowed to stand in the dark for 10 min.

### 2.3. Selection of Strains of Endophytic Fungi from Papaya with Antifungal Activity

A preliminary screening of the antifungal activity was carried out by using the plate confrontation method. Due to the massive variety of endophytic fungi to be determined, the first screening made use of a five-point confrontation culture method: first, a fungal cake of a pathogen was positioned at the core of the PDA medium; at the same time, 4 exceptional endophytic fungi with an equal diameter were inoculated at 4 locations equidistant from the pathogen. The corresponding colony name, date, etc., were marked on the back of the plate. Meanwhile, the antagonistic effects of endophytic fungi on the PDA plates, the presence or absence of an antifungal zone, hyphal atrophy, and deformity, etc., were assessed and recorded. If positive phenomena occurred, they were used as a preliminary basis for judging whether the endophytic fungi had antagonistic and antifungal activity.

The secondary screening utilized a confrontation culture method with two points. Endophytic fungi and pathogenic fungi were inoculated on opposite sides of a straight line, equidistant from the center point of the PDA plates. Blank controls were set up using only pathogenic bacteria, with the experiment being repeated three times. The plates were incubated at a constant temperature of 28 °C for approximately 3–5 days, and the radial growth of pathogenic bacteria colonies was measured using a cross method. The inhibition rate of the endogenous fungi in opposition to pathogenic fungi was calculated as follows:

Inhibition rate (%) = [(Control group pathogen radius − Treatment group pathogen radius)/(Control group pathogen radius)] × 100%

The test data were taken as the average of the 3 replicates.

### 2.4. Detection of the Control Effect of Endophytic Fungi from Papaya on Colletotrichum gloeosporioides

Preparation of the suspension: *Colletotrichum gloeosporioides* was inoculated into an inclined tube containing PDA; after the fungus grew over the slope, it was rinsed with sterile water, and inoculation loops were used to scrape and gather spores, and then place them in sterile distilled water. The samples were filtered with sterile double-layer gauze to reap a spore suspension of anthrax. The spore suspension was adjusted to the favored concentration using a hemocytometer.

Preparation of the test strain Y17 (*Penicillium rolfsii*, MT729953) at 99%: The fungal samples were cultured in a suspension; the endophytic fungus Y17 was inoculated in an inclined tube containing PDA, as in the preceding operations, and a spore suspension of the endophytic fungus Y17 was acquired. The spore suspension was adjusted to the favored concentration using a hemocytometer.

Preparation of a fermentation broth for test strain Y17: The concentration of the endogenous fungus Y17 was adjusted to 10^6^ CFU/mL with the aid of a blood count plate for inoculation in the course of fermentation. Then, 300 mL of PDB medium was added to a 500 mL Erlenmeyer flask, and the inoculum of the endophytic fungus Y17 was 1.0% (*v*/*v*). The system was set to 28 °C and 160 r/min, and fermentation took place in a shaker for 15 d. Vacuum filtration was carried out after the sample was removed, and then the mycelium and fermentation broth were filtered and separated. Then, the fermentation broth was filtered through a 0.45 μm filter membrane to acquire the pattern solution, and the pattern sample was kept at 4 °C for storage.

Pretreatment was carried out in accordance with the method described in [35]. Whole papaya fruits of essentially equal size and maturity were selected, washed with 75% ethanol solution, washed with sterile water, and dried. The test group was immersed in the fermentation broth of the endophytic fungus Y17 for 5 min, and the control group was immersed in sterile water for 5 min. After the fruits were dried, a stab-wound inoculation technique was used to make a small gap with a diameter of about 4 mm and a depth of about 20 mm at the equator of the papaya fruit, and 20 μL of *Colletotrichum gloeosporioides* suspension was inoculated at a concentration of 10^6^ CFU/mL at the wound site. The control group and the treatment group each consisted of 10 handled fruits, and the experiment was repeated 3 times. Observations were made according to the method described by Li et al. [36]. The disease index and relative prevention and treatment effects were calculated as follows:

Disease index = [Σ(Disease grade × Number of lesions)/(Highest disease grade × The total number of lesions was investigated)] × 100%

Relative control effect = [(Disease index of control group − Treatment group disease index)/Disease index of control group] × 100%

### 2.5. Determination of Defense Enzyme Activity and Related Indexes in the Fruit after Treatment with Endophytic Fungi

All the papaya fruits that were used were labeled, numbered, and saved in a mild incubator at a consistent temperature of 25 °C. The fruits were assessed every 24 h. Two fruits were randomly chosen from every cure group; the pulp tissue within 2 cm of the border of the diseased and wholesome tissue was taken as the core of the inoculation point, and a combination of the two inoculation factors was used as one repetition. The above test was repeated 3 times.

The procedure for extracting the crude enzyme solution was as outlined in [36] and underwent minor modifications. The precise steps were as follows: 1 g of pulp tissue was accurately weighed and placed in a preprepared, precooled mortar. Subsequently, a 50 mmol/L, pH 7.4 PBS buffer at a ratio of 1:9 (*w*/*v*) was introduced into an ice bath, and the mixture was rapidly ground. Following this, the combination was centrifuged at 10,000 g/min at 4 °C for 20 min, yielding the extracted supernatant; this constituted the crude enzyme solution for evaluating the protective enzyme activity. Catalase (CAT) was quantified using the ammonium molybdate method [37], peroxidase (POD) was quantified using the guaiacol method [38], and superoxide dismutase (SOD) was quantified using the hydroxylamine approach [39]. The malondialdehyde (MDA) content was measured with the thibabituric acid (TBA) method [40]. The MDA content is expressed in nmol/g based on the fresh weight, while the total antioxidant capacity is expressed as the homogenized protein concentration in mmol/g prot. The determination of the total antioxidant ability (T-AOC) was conducted using the FRAP technique [41]. For the preparation of the sample for assessing the total antioxidant potential, 0.25 g of pulp tissue was accurately weighed and placed in a precooled mortar that was prepared in advance, and a 50 mmol/L pH 7.4 phosphate buffer was added in a ratio of 1:4 (*w*/*v*) under ice bath conditions for rapid grinding. The resulting solution was centrifuged at 10,000 g/min and 4 °C for 5 min, yielding the extracted supernatant, which served as the sample liquid for assessing the total antioxidant capacity. The enzyme activity within the sample was quantified as the fresh weight in U/g per gram of pulp tissue. The absorbance of the sample solution was measured at a wavelength of 593 nm [42]. All data were collected and derived from the average of three measurements.

### 2.6. Construction of a Phylogenetic Tree of the Active Strains

The ITS sequences of the endophytic fungi that were sequenced, were searched and compared in the NCBI database using the BLAST program, and related species’ sequences were downloaded. MEGA7.0 software [11] was applied to construct a phylogenetic tree, using the neighbor-joining method; the bootstrap test value was ≥50%, with a total of 1000 repetitions [43].

### 2.7. Statistical Analysis

The IC50 value was determined through linear regression analysis, by employing SPSS 19.0. SAS 9.4 software was utilized for the statistical assessment of the original data, where a significance level of *p* < 0.05 was considered statistically significant. Excel 2013 and the GraphPad 8.0 software were employed for data processing and graph creation.

## 3. Results

### 3.1. Isolation of Endophytes

Endophytic fungi were meticulously isolated and cultivated on PDA, following the thorough surface sterilization of the papaya tissues. Subsequently, colonies emerged on the PDA three days later. A total of 131 endophytic fungi (Table S1) were isolated from 702 segments of stems, leaves, and fruits. Notably, the highest isolation rate was observed for the fruits, followed by the leaves and stems (Table 1), showcasing a diverse array of endophytic fungal species associated with papaya.

### 3.2. Identification

PCR Amplification and Sequencing. The entire DNA of the 131 strains isolated from papaya was extracted and utilized as a template for PCR amplification. Employing ITS1F and prs4 as primers for the PCR amplification of ITS sequences, the molecular weight of the PCR products for each strain was determined to be within the range of 500–750 bp, through gel electrophoresis (Figure S1). This aligned with the molecular weight standard for the ITS (internal transcribed spacer) and could, thus, be employed for the subsequent sequencing and molecular identification of the strains.

Based on the BLAST alignment of the 131 sequences of endophytic fungi using the NCBI database, these fungi were systematically classified into 20 genera, including *Phoma* spp., *Phyllosticta* spp., *Aspergillus* spp., *Cerrena* spp., *Fusarium* spp., *Candida* spp., and *Penicillium* spp., among others. In terms of the relative frequency (RF) of the isolated endophytic fungi, *Aspergillus* spp. (47.33%) and *Colletotrichum* spp. (14.50%) emerged as the predominant groups within the papaya ecosystem, while other groups exhibited frequencies below 10% (Table 2). As the dominant genus in papaya, *Aspergillus* spp. was successfully isolated from the stems, leaves, and fruits. However, the other dominant genus, *Colletotrichum* spp., was not isolated from the fruits. This observation may be attributed to variances in physiological structures and nutrient compositions among distinct tissue components, which create unique microenvironments that influence the infection, growth, and distribution of endophytic fungi.

### 3.3. Screening of Antioxidant Strains

#### 3.3.1. Radical Scavenging Assays

DPPH exhibits remarkable stability in natural solvents and is a commonly employed radical for evaluating the antioxidant potential of various organic samples [29]. When ethyl acetate extracts of endophytic fungi were employed at a concentration of 100 g/mL for the initial screening, seven strains demonstrated free-radical-scavenging rates ranging between 53.90 and 82.72%, as shown in Table 3. These seven strains exhibited decreased free-radical-scavenging rates compared with that of the positive control (VC), which had a scavenging rate of 97.85%, and BHT, which had a scavenging rate of 88.46%. Subsequently, we revealed a direct correlation between the scavenging rates and increased ethyl acetate concentration within the range of 6.25–100 mg/L. The IC50 values of the seven strains ranged from 19.72 to 84.06 mg/L. Among them, the endophytic fungus Y17, which was extracted from papaya leaves, demonstrated outstanding DPPH radical-scavenging abilities.

The ABTS^+^ cation, which is generated through the oxidation of ABTS, is a stable free radical that exhibits a blue–green hue when dissolved in a solution. Upon interaction with antioxidant substances, the color of the ABTS solution lightens, and this is accompanied by a reduction in absorption values [44]. Analogously to the outcomes of the DPPH free-radical screening test, the same seven strains exhibited ABTS free-radical-scavenging rates exceeding 50%, ranging from 66.16 to 91.83%. These strains were identified by utilizing ethyl acetate extracts of endophytic fungi at a concentration of 100 g/mL for the initial screening, as shown in Table 4. This finding indicated that these seven strains performed commendably in various ethyl acetate extracts through an in vitro assessment of their antioxidant activity. The ABTS free-radical-scavenging rates of the seven strains exhibited a notable reduction when compared with the highly commendable 99.60% scavenging rate of vitamin C (VC) and that of BHT, which demonstrated a scavenging rate of 93.60%. Within the concentration range of 6.25–100 mg/L of ethyl acetate, the IC50 values of the seven strains ranged from 14.34 to 64.63 mg/L. Notably, the endophytic fungus Y17 extracted from papaya leaves exhibited exceptional ABTS radical-scavenging capabilities.

#### 3.3.2. Reducing Power Assay

**Figure 1:**
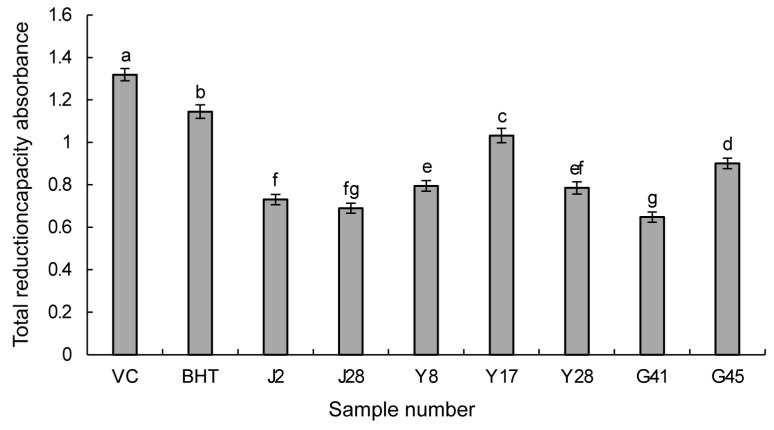
Determination of the total reduction ability of ethyl acetate extracts from endophytic fungi. The data in the figure are the average of three repetitions. VC and BHT were used as positive controls, while the remaining 7 strains served as test samples. Different lowercase letters indicate significant differences (*p* < 0.05).

Antioxidants have the capacity to donate electrons through their own reduction to scavenge free radicals. The higher the reducing power of antioxidants, the greater their antioxidant activity. As shown in Figure 1, the higher the bar chart, the higher the absorption value, and the stronger the reducing capacity of the sample. When the absorbance values of the seven screened strains were assessed, Y17 exhibited the highest total reducing power, followed by G45, Y8, Y28, J2, J28, and G41. Although the power was slightly lower than that of BHT and the natural antioxidant VC (Figure 1), these results align with the DPPH and ABTS radical scavenging assays, confirming the outstanding antioxidant activity of the ethyl acetate extract from Y17. Consequently, further explorations and applications of this strain and its metabolites are warranted.

### 3.4. Morphological Identification of Y17 and the Construction of a Phylogenetic Tree

**Figure 2:**
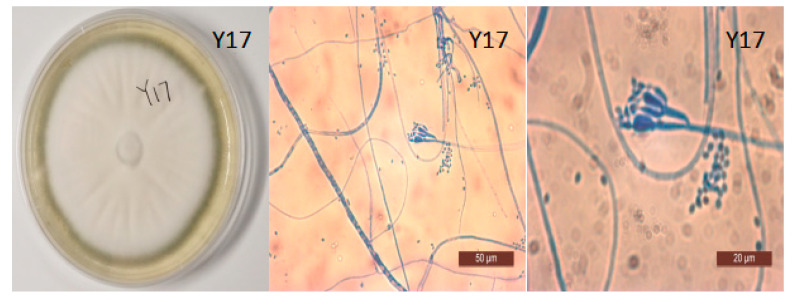
Plate colony morphology and morphology of the conidia of the endophytic fungus Y17.

**Figure 3:**
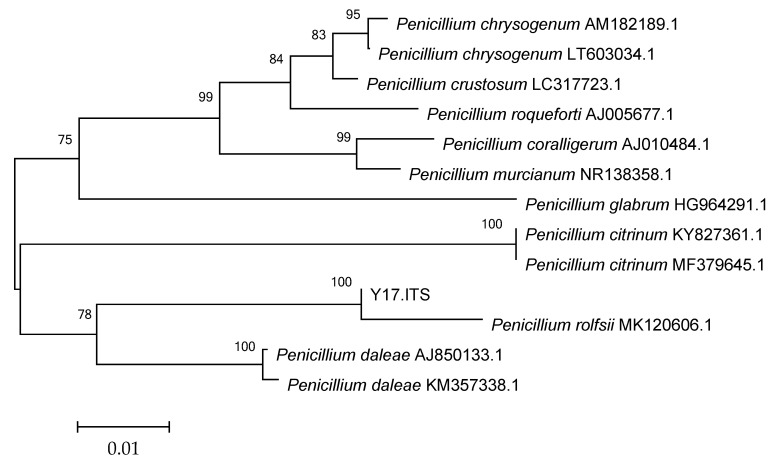
Phylogenetic tree of the endophytic fungus Y17 and related species in papaya.

The ITS sequences of strain Y17 (Appendix S1) were subjected to a comparison in the NCBI database, using the BLAST program. Subsequently, the ITS sequence information on the strains displaying the highest similarity and those belonging to related species were retrieved and stored. MEGA7.0 software was then employed to construct a phylogenetic tree, thus further elucidating the species. In the phylogenetic tree, which was based on the ITS sequences of 13 strains, Y17 and *Penicillium rolfsii* (MK 120606.1) were clustered together on the same branch. According to an analysis of the ITS sequences and an assessment of the colony characteristics on PDA (Figure 2), Y17 was preliminarily identified as *Penicillium rolfsii* (Figure 3).

### 3.5. Screening of Strains with Antifungal Activity

In this experiment, the antifungal efficacy of various strains was assessed using the plate confrontation method. Following an initial screening at five points and subsequent double screening, 13 endophytic fungal strains from papaya exhibiting inhibitory activity against the test fungi used in this study were identified. These represented 9.92% of the total isolated strains. As detailed in Table 5, among the endophytic fungi demonstrating antifungal activity, strains Y17 (54.09%), J16 (58.09%), G16 (3.82%), and G45 (52.64%), exhibited exceptional activity against *Colletotrichum gloeosporioides* in papaya, with inhibition rates exceeding 50%. Moreover, Y17, J16, and Y28, displayed effective inhibition against stem rot involving *Neoscytalidium dimidiatum*, while Y28, G39, and J8 demonstrated inhibition rates exceeding 50% against *Pestalotiopsis* spp. and exhibited resilience against *Fusarium oxysporum*. These strains also exhibited robust inhibitory activity against *Botryodiplodia theobromae*. Among the fungal strains, *Aspergillus* spp. accounted for a significant proportion, and seven strains were identified (J16, J17, Y23. Y28, G5, G16, G45). Additionally, there were two strains of *Penicillium* spp. (Y17, Y8), as well as one strain each of *Fusarium* spp., *Pythium* spp., *Pleurotus* spp., and *Candida* spp. Notably, as shown in Table 5, *Penicillium* spp. strain Y17 displayed specific antifungal activity against four pathogens, excluding *Fusarium oxysporum*, showcasing a broader antifungal spectrum. Y17 exhibited the most potent bacteriostatic activity against *Colletotrichum gloeosporioides* penz., achieving 54.09% inhibition (Table 5).

### 3.6. Detection and Control Effects of the Active Antifungal Strain Y17 on Colletotrichum gloeosporioides

**Figure 4:**
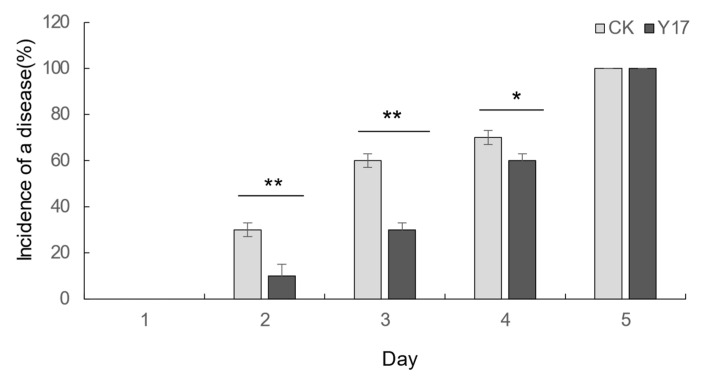
Changes in the incidence of *Colletotrichum gloeosporioides* over time. The values in the figure are the mean ± standard deviation of three replicates. The asterisks indicate a significant difference (* *p* < 0.05, ** *p* < 0.01), according to a *t*-test. CK: *Colletotrichum gloeosporioides* was added after 5 min of immersion in sterile water. Y17: The fermentation solution of the endophytic fungus Y17 was added after 5 min of immersion, and then *Colletotrichum gloeosporioides* was added.

**Figure 5:**
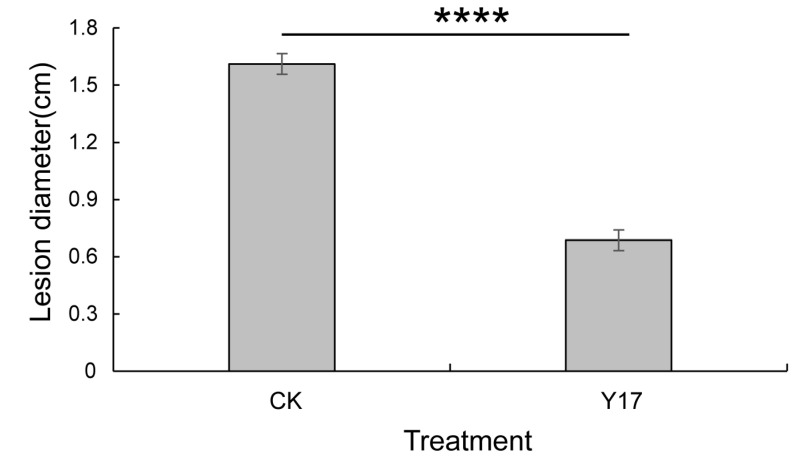
Spot area of *Colletotrichum gloeosporioides* in papaya fruit. The values in the figure are the mean ± standard deviation of three replicates. The four asterisks indicate a very significant difference (*p* < 0.001), according to a *t*-test. CK: *Colletotrichum gloeosporioides* was added after 5 min of immersion in sterile water. Y17: The fermentation solution of the endophytic fungus Y17 was added after 5 min of immersion, and then *Colletotrichum gloeosporioides* was added.

**Figure 6:**
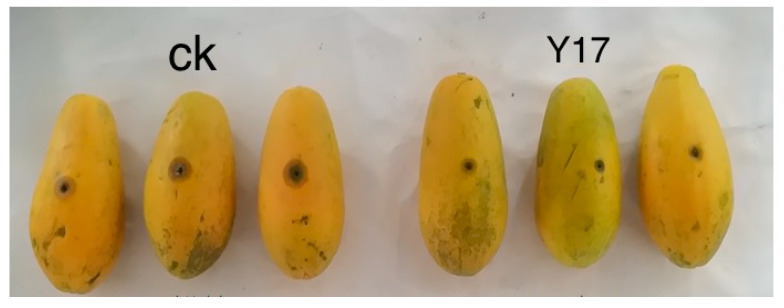
The control efficacy on papaya fruit infected with *Colletotrichum gloeosporioides*. CK: *Colletotrichum gloeosporioides* was added after 5 min of immersion in sterile water. Y17: The fermentation solution of the endophytic fungus Y17 was added after 5 min of immersion, and then *Colletotrichum gloeosporioides* was added.

The strain Y17 (*Penicillium rolfsii*) showed good broad-spectrum antibacterial activity in the plate confrontation test, and the secondary metabolites of Y17 also showed good antioxidant activity in the previous antioxidant activity screening. These results indicated that the endophytic fungi isolated from papaya leaves (Y17) had good biological activity. After comprehensive consideration, it was decided to use the fermentation broth of the endophytic fungus Y17 to test the control effect of *Colletotrichum gloeosporioides* from papaya fruits and to analyze its potential as a biocontrol strain. It was found in the experiment that both the control group and the treatment group had disease at the site of the stab-wound inoculation 5 days after the inoculation. When comparing the incidence in the control group and the treatment group, it was found that the incidence rate of the Y17 treatment group significantly slowed down compared with that of the control group (Figure 4). Following the 5-day inoculation with *Colletotrichum gloeosporioides* pathogens in papaya, we measured the extent of the lesions at the inoculation site. A significant disparity in the lesion areas emerged between the Y17 treatment group and the control group (*p* < 0.05). The lesion area decreased from 2.03 to 0.37 cm^2^ (Figure 5), demonstrating a substantially lower incidence rate in the Y17 group compared with that in the CK group (Figure 6). This observation suggested a distinct inhibitory effect on *Colletotrichum gloeosporioides* in papaya fruit.

### 3.7. Effect of the Active Antifungal Strain Y17 on the Antioxidant System of Papaya Fruit

**Figure 7:**
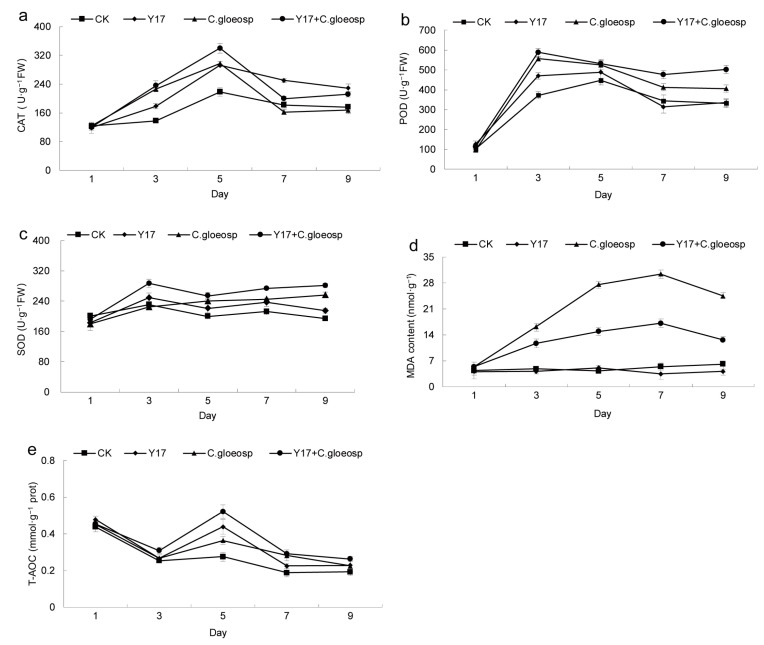
Effects of strain Y17 on the antioxidant system of papaya fruit. (**a**) changes in the activity of catalase (CAT); (**b**) changes in the activity of peroxidase (POD); (**c**) changes in the activity of superoxide dismutase (SOD); (**d**) changes in the content of malondialdehyde (MDA); (**e**) changes in the total antioxidant capacity (T-AOC). The experimental layout involved four treatment groups with 10 fruits in every treatment group, and the experiment was repeated three times after pretreatment of the papaya fruits, as described in above. The papaya fruits were handled as follows: treatment (CK) involved sterile water soaking treatment as a control; treatment (Y17) involved immersion in the fermentation broth of the endophytic fungus Y17; treatment (*C. gloeosp*) involved stab-wound inoculation with *Colletotrichum gloeosporioides* penz.; treatment (Y17 + *C. gloeosp*) involved the endophytic fungus Y17 being soaked in fermentation broth for 5 min and inoculated with *Colletotrichum gloeosporioides*.

Catalase (CAT), superoxide dismutase (SOD), and peroxidase (POD) constitute integral components of a plant’s antioxidant enzyme system [45]. Elevated CAT enzyme activity serves to mitigate peroxidative damage induced by reactive oxygen species. In this study, as illustrated in Figure 7a, all of the treatment groups exhibited an initial increase followed by a decline, with each group attaining peak enzyme activity on the fifth day. The activity of the enzymes in the Y17 + *C. gloeosp* treatment group surpassed that in the CK group by 1.55 times, signifying a significant disparity between the two groups (*p* < 0.05). Subsequently, while the activity in the Y17 + *C. gloeosp* treatment group began to decrease, it consistently remained higher than that of the CK group. Following the peak enzyme activity, there was a reduction in the activity of the enzymes in both the Y17 and Y17 + *C. gloeosp* groups after treatment with the fermentation broth, and that of the Y17 group declined more slowly than that of the *C. gloeosp* group. This outcome suggested a discernible elevation in the CAT enzyme activity in the fruit following treatment with the fermentation broth.

The peroxidase (POD) enzyme is a pivotal component within the intricate antioxidant system of plants. Its expression is most notably manifested during the early stages of adversity or aging, demonstrating a robust shielding effect [46,47]. As illustrated in Figure 7b, the activity of POD exhibited a significant elevation across all treatment groups on the third day. Notably, the fruits in the *C. gloeosp* and Y17 + *C. gloeosp* groups attained peaks in their activity, registering 1.49 times and 1.58 times that of the CK group, respectively. A substantial difference was evident between these groups (*p* < 0.05). In the subsequent days, the enzyme activity in the *C. gloeosp* and Y17 + *C. gloeosp* treatment groups underwent a moderate decline, but remained higher than that observed in the CK group. The fermentation activity may have contributed to the augmented POD enzyme activity during infection with *Colletotrichum gloeosporioides*. Remarkably, the fruit itself demonstrated resistance to potential infection and colonization by *Colletotrichum gloeosporioides*.

Numerous studies have asserted that the resilience of plants to adverse conditions is intricately linked to the SOD activity levels under unfavorable circumstances [48]. As illustrated in Figure 7c, the SOD activity of the CK group exhibited minimal variation from day 1 to day 9, whereas that of the Y17 treatment group saw an increase on the third day, followed by more gradual alterations. The enzyme activity in the *C. gloeosp* treatment group consistently and steadily increased, but it remained lower than that of the Y17 + *C. gloeosp* treatment group. By the third day, the enzyme activity in the Y17 + *C. gloeosp* group experienced a substantial surge, with a faster rate of change and activity levels surpassing those of the other groups. By the ninth day, the enzyme activity in the Y17 + *C. gloeosp* group reached 1.45 times that of the CK group, marking a significant difference (*p* < 0.05) across all treatment groups at this juncture. Pretreatment with the Y17 fermentation broth demonstrated an ability to mitigate the damage inflicted by *Colletotrichum gloeosporioides* fungi, by enhancing the SOD enzyme activity when the fruit was under stress due to *Colletotrichum gloeosporioides* infection.

Malondialdehyde (MDA) is a primary byproduct of lipid peroxidation in plants. The quantification of MDA indirectly serves as a reflective measure of the extent of fruit damage resulting from peroxidation processes [49]. As depicted in Figure 7d, the MDA content in the fruits of the *C. gloeosp* and Y17 + *C. gloeosp* treatment groups, which were subjected to *Colletotrichum gloeosporioides* inoculation, displayed a significant increase over the observation period. By the seventh day, the MDA content of the *C. gloeosp* and Y17 + *C. gloeosp* groups was 5.62 times and 3.16 times higher than that of the CK group, respectively. Remarkably, the MDA content of the Y17 + *C. gloeosp* treatment group was substantially lower than that of the *C. gloeosp* group (*p* < 0.05). These findings underscored the compromised metabolic resilience of papaya fruits in responding to reactive oxygen species, leading to an escalation in membrane lipid peroxidation. Following treatment with the Y17 fermentation broth, the accumulation of MDA in the papaya fruits inoculated with pathogens experienced a significant reduction. This outcome indicated the mitigation of the degree of membrane lipid peroxidation during this period. Notably, the fermentation liquid derived from the endophytic fungi Y17 exhibited a preventive effect against fruit membrane lipid peroxidation to a considerable extent, thereby enhancing the fruit’s resistance to disorders.

A plant’s antioxidant system comprises two main constituents [50]: the enzymatic system and the nonenzymatic system. The measured CAT, POD, and SOD activity, as well as similar parameters that were mentioned earlier, are indicative of the enzymatic protection system [51]. Conversely, the T-AOC adeptly reflects the holistic electrical balance within a plant’s antioxidant system. It serves as a sophisticated metric, epitomizing the comprehensive antioxidant prowess intrinsic to the plant itself. As delineated in Figure 7e, the trajectory of the overall antioxidant capacity in the fruits illustrated a consistent decline in the CK group over time. In contrast, the different treatments manifested distinctive phases of elevation on the fifth day. Notably, the Y17 + *C. gloeosp* treatment group exhibited a remarkable surge to 1.92 times that of the CK group. Subsequently, each group unexpectedly regressed to the baseline of the control group. This phenomenon may be attributed to an upsurge in free radicals resulting from respiration and pathogen infection on the fifth day, coinciding with fruit ripening. This activated the fruit’s inherent defense mechanisms, triggering an influx of reactive oxygen to combat the *Colletotrichum gloeosporioides* invasion. The augmentation of the fruit’s overall antioxidant capacity acted as a deterrent, mitigating the deleterious effects of excessive reactive oxygen free radicals. Regardless of *Colletotrichum gloeosporioides* inoculation, the fruit’s antioxidant capacity was significantly higher in the fermentation-broth-treated group compared with that of the CK group on the fifth day (*p* < 0.05). This implied that the fermentation broth induced a marked enhancement in the fruits’ T-AOC, showcasing its efficacy in fortifying antioxidant defenses.

## 4. Discussion

In this experiment, the surface disinfection prerequisites involved a 75% ethanol solution for 75 s and a 2.6% NaClO solution for 150 s. Based on the results of colony growth, the separation effect was found to be optimal. A total of 131 endophytic fungi were isolated from 729 tissue samples, resulting in an isolation rate of 18.66%. Notably, the highest isolation rate was observed in the fruit samples, followed by the leaves and stems. This suggested that the level of infection with endophytic fungi in the fruits was relatively high, which was likely due to variations in tissue composition and nutrient content, resulting in the formation of distinct microenvironments within the host plants and subsequently influencing the contamination and distribution of endophytic fungi. The 131 strains of endophytic fungi were classified into 20 different genera, based on their morphological traits in analyses of PDA and ITS sequences. Specifically, 9 genera were isolated from the stems, 10 genera were isolated from the leaves, and 15 genera were isolated from the fruits. Among these, the dominant groups were *Aspergillus* and *Echinococcus*, which together accounted for more than 50% of the total number of strains. As the predominant species, *Echinococcus* was not found within the fruits; however, *Colletotrichum gloeosporioides* penz., which is a member of *Echinococcus*, was isolated from both the leaves and stems. This occurrence might have been linked to the selection of the testing materials. Furthermore, several less common species were identified, including *Cerrena* spp., *Diaporthe* spp., *Phialemonium* spp., and *Talaromyces* spp. Consequently, the allocation of endophytic fungi across distinct tissue components of the same host plant exhibited a specific tissue specificity, which is in alignment with prior research [9,52].

Ethyl acetate extract from endophytic fungi at a concentration of 100 mg/L was selected for the initial screening, and by employing DPPH and ABTS free radical scavenging assays, we identified seven strains with a scavenging rate exceeding 50%. Notably, J2, Y8, Y17, and G41 were classified under *Penicillium* spp., while G45 and Y28 were attributed to *Aspergillus* sp., and J28 was attributed to *Phyllosticta* spp. From the pool of strains exhibiting antioxidant activity, two were isolated from papaya stems, three were isolated from papaya leaves, and two were isolated from papaya fruits. This suggested that papaya hosts distinct strains with heightened antioxidant capabilities; they are distributed across its various components and among different species. In the concentration range of 6.25–100 mg/L, an escalation in the sample concentration of the ethyl acetate extract from each strain correlated positively with increased scavenging rates for ABTS and DPPH free radicals, indicating a dose–effect relationship. This finding aligns with the outcomes in the investigation by Zeng et al. involving the in vitro antioxidant activities of endophytic fungi isolated from liverwort (*Scapania verrucosa*) [31]. Moreover, for the same plant, the distribution of endophytic fungi within the host exhibited tissue specificity. Zhang et al. gathered 625 endophytic fungi from diverse parts of oil tea (*Camellia oleifera*) plants, including the stems, leaves, fruits, and bark [53]. Their study corroborated that the distribution of endophytic fungi varies across different plant parts.

However, the free-radical-scavenging rates of each strain were lower than those of the positive control antioxidants (VC and BHT) at equivalent concentrations. Upon comparing the IC50 values of the samples, it was established that the half-inhibitory concentrations of the two free radicals in Y17 were less than 20 mg/L, indicating significantly stronger antioxidant activity compared with that of the other strains. Furthermore, the ethyl acetate extract of Y17 exhibited the highest antioxidant activity, according to the assessment of its overall reduction capabilities with the iron–potassium cyanide method, further substantiating its potential as a natural antioxidant. This study focused solely on ethyl acetate extracts derived from papaya’s endophytic fungi, potentially omitting some active strains due to the limited solubility of antioxidant components in ethyl acetate. Future research should explore alternative extractants, such as petroleum ether, methanol, and n-butanol, to comprehensively analyze the distribution of antioxidant components. Optimizing the fermentation conditions is essential for enhancing the antioxidant capacity of stress metabolites [54].

Studies have revealed that plant endophytic fungi primarily manage plant diseases through three mechanisms: (1) competing nutritionally with pathogenic fungi, (2) secreting antifungal substances, and (3) inducing plant resistance or promoting plant growth to control plant diseases [55]. Moreover, when utilizing endophytic fungi as biocontrol agents, these mechanisms often exhibit synergistic effects, resulting from the combined influence of multiple factors. This investigation involved 13 fungal strains demonstrating antifungal activity against at least one tested pathogenic fungus in plants (Table 5). Among these, seven strains of *Aspergillus* spp. and two strains of *Penicillium* spp. demonstrated superior antifungal activity. Numerous investigations have substantiated that *Aspergillus* spp. and *Penicillium* spp. exhibit effective antifungal properties and can produce bioactive substances. For instance, Qin et al. found that *Aspergillus terreus*, an endophytic fungus found in cactus plants, yields broad-spectrum and highly active antifungal compounds, exerting inhibitory effects on 21 distinct pathogenic microorganisms [56]. Fu et al. revealed that a strain of the endophytic fungus, *Penicillium polonicum*, displayed robust bacteriostatic activity against two plant pathogens causing *Fusarium* and *Verticillium wilt* in cotton. Upon treatment of the fermentation broth with protease, the bacteriostatic activity disappeared, leading to the speculation that the active bacteriostatic ingredient in the fermentation broth belonged to the protein category [57]. One strain of the endophytic fungus identified as *Penicillium rolfsii*, which was designated as Y17, exhibited enhanced bacteriostatic activity against four types of indicator fungi, excluding watermelon Fusarium wilt, thus being of value for further research. 

The outcomes of the plate confrontation test in this study revealed that the endophytic fungus strain Y17 (*Penicillium rolfsii*) inhibited the growth of the mycelium of *Colletotrichum gloeosporioides* in papaya. It is presumed that there is a certain level of competition and antagonism between this strain and *Colletotrichum gloeosporioides* in papaya. In the evaluation of the fruit’s control effect, following the application of the Y17 fermentation broth, there was a substantial reduction in the areas affected by *Colletotrichum gloeosporioides* spots at the site of inoculation. Simultaneously, the progression of the disease was notably slowed down. An initial speculation arose regarding the potential presence of antifungal agents within the fermentation broth, possibly impacting the growth and reproduction rate of the *Colletotrichum gloeosporioides* pathogen. Under the influence of adverse external factors, plants tend to bolster their resistance by enhancing enzyme activity against external stimuli. In the realm of plant physiology, the pivotal antioxidant enzymes POD, SOD, and CAT play a crucial role in the steady augmentation and development of the plant [58]. MDA is the primary byproduct of cellular membrane lipid peroxidation, while the T-AOC substantiates the comprehensive antioxidant capacity of the fruit. The assessment of alterations in the activities of these three antioxidant enzymes, as well as the levels of MDA and T-AOC, in the pulp tissue, indirectly signified whether the treatment of the fruits with the fermentation broth of the antifungal strain Y17 induced an enhancement in their resistance to pathogenic invasion. The determination of the activity of protective antioxidant enzymes revealed that the group subjected to *Colletotrichum gloeosporioides* penz. fungi after pretreatment with the Y17 fermentation broth exhibited heightened activity of three protective antioxidant enzymes, namely CAT, POD, and SOD, in comparison with those that were solely treated with *Colletotrichum gloeosporioides*. Notably, the T-AOC exhibited a substantial increase by the fifth day (*p* < 0.05). These findings corroborate the notion that certain metabolites present in the fermentation broth potentially stimulate the ability of papaya fruit to fortify its resilience against pathogen intrusion. The present discovery aligns with the findings by Shi et al., who effectively stimulated the enhancement of disease resistance in papaya fruit by elevating the overall phenol content, as well as the activities of crucial protective enzymes, such as CAT and POD. This improvement was observed after treatment with the endogenous papaya fungus MGY2 [59]. Notably, the MDA content, which is indicative of membrane lipid peroxidation, exhibited a significant decrease compared with that of the group that was solely inoculated with *Colletotrichum gloeosporioides* (*p* < 0.05). Similar outcomes were reported by Zhu et al., who utilized endophytic actinomycetes to induce resistance against leaf blight due to *Colletotrichum gloeosporioides* in apples [60]. It was revealed that the endophytic fungus strain Y17 (*Penicillium rolfsii*) exerts a distinct regulatory effect on *Colletotrichum gloeosporioides* in papaya fruit, suggesting its potential for further refinement and application as a biocontrol agent. It is important to note that, in this investigation, only the fermentation broth from the active strain was employed to assess the control effect, and an examination of the colonization and the detection of live fungi were omitted. Future studies should incorporate live fungi as inducers to intricately investigate their role in reinforcing papaya fruits’ disease prevention mechanisms.

## 5. Conclusions

In this study, the presence of isolated and recognized endophytic fungi from various parts of papaya were investigated, thereby expanding the reservoir of cultivable plant endophytic fungi. The antioxidant capacity was evaluated according to DPPH and ABTS assays and the total reducing capacity. It was found that *Penicillium rolfsii* (Y17) isolated from endophytic fungi in papaya leaves had strong antioxidant capacity. Concurrently, it was observed that the active strain Y17 significantly inhibited the infection and colonization of the *Colletotrichum gloeosporioides* pathogen in papaya fruit wounds, effectively reducing the disease rate. Furthermore, it enhanced the activity of CAT, POD, SOD, and other protective enzymes within the antioxidant enzyme system of papaya fruit, mitigating the rate of MDA accumulation and augmenting the overall antioxidant capacity of the fruit itself. To a certain extent, it improved the fruit’s resistance to pathogen invasion and colonization. The Y17 strain, which was isolated from papaya leaves, exhibited antioxidant activity, and demonstrated control efficacy against *Colletotrichum gloeosporioides* in papaya fruit.

## Figures and Tables

**Table 1:** Rate of isolation of endophytic fungi from papaya.

	Stem	Leaf	Fruit	Summation
Number of fungal strains	31	45	55	131
Isolation frequency, %	12.76	18.52	22.63	18.66

**Table 2:** The genera of endophytic fungi in papaya fruits, stems, and leaves.

Endophytic Fungi Genera	Separation Tissue			Relative Separation Frequency, %
	Stem, %	Leaf, %	Fruit, %	
*Aspergillus* spp.	8.40	16.03	22.90	47.33
*Colletotrichum* spp.	5.34	9.16	0	14.50
*Candida* spp.	1.53	2.29	4.58	8.40
*Cladosporium* spp.	2.29	0	3.82	6.11
*Penicillium* spp.	2.29	1.53	2.29	6.11
*Fusarium* spp.	1.53	1.53	0.76	3.82
*Phoma* spp.	0.76	0.76	0	1.53
*Phyllosticta* spp.	0.76	0.76	0	1.53
*Trichoderma* spp.	0	0.76	0.76	1.53
*Talaromyces* spp.	0	0.76	0.76	1.53
*Diaporthe* spp.	0	0.76	0	0.76
*Phialemonium* spp.	0	0	0.76	0.76
*Neofusicoccum* spp.	0	0	0.76	0.76
*Sporothrix* spp.	0	0	0.76	0.76
*Cerrena* spp.	0.76	0	0	0.76
*Wallemia* spp.	0	0	0.76	0.76
*Gibellulopsis* spp.	0	0	0.76	0.76
*Phlebia* spp.	0	0	0.76	0.76
*Alternaria* spp.	0	0	0.76	0.76
*Daldinia* spp.	0	0	0.76	0.76

Note: The relative isolation frequency was the ratio of the number of strains in a certain group to the total number of strains isolated.

**Table 3:** Scavenging rate of DPPH free radicals by the endophytic fungi from papaya.

Sample Name	DPPH Scavenging Rate, %, at Different Concentrations of Samples					IC50 Value, mg/L
	6.25 mg/L	12.5 mg/L	25 mg/L	50 mg/L	100 mg/L	
VC	30.37 ± 1.04 ^a^	43.43 ± 0.91 ^a^	70 ± 0.68 ^a^	96.63 ± 0.16 ^a^	97.85 ± 0.04 ^a^	12.55 ± 0.0004 ^ e ^
BHT	17.91 ± 0.81 ^b^	38.92 ± 0.35 ^b^	71.4 ± 0.27 ^a^	83.68 ± 0.60 ^b^	88.46 ± 0.26 ^b^	16.01 ± 0.0031 ^ e ^
Y17	17.17 ± 0.38 ^b^	38.22 ± 0.29 ^b^	63 ± 0.45 ^b^	70.88 ± 0.84 ^c^	82.72 ± 0.68 ^c^	19.72 ± 0.0052 ^ e ^
Y28	3.03 ± 0.48 ^d^	10.8 ± 0.73 ^d^	28.49 ± 0.39 ^c^	52.31 ± 0.24 ^d^	71.4 ± 0.13 ^d^	49.22 ± 0.0016 ^ d ^
J28	9.58 ± 0.85 ^c^	19.35 ± 0.91 ^c^	29.63 ± 0.76 ^c^	43.88 ± 0.30 ^f^	69.03 ± 0.22 ^e^	54.32 ± 0.0027 ^ d ^
G45	3.48 ± 0.27 ^d^	9.36 ± 0.46 ^d^	29.41 ± 0.23 ^c^	36.63 ± 0.48 ^g^	65.63 ± 1.00 ^e^	64.44 ± 0.0033 ^ c ^
Y8	0.92 ± 0.74 ^d^	6.81 ± 0.43 ^e^	25.27 ± 0.75 ^d^	36.39 ± 0.95 ^e^	53.9 ± 0.1 ^g^	72.07 ± 0.0050 ^ b ^
J2	1.44 ± 0.45 ^d^	10.06 ± 0.15 ^d^	20.09 ± 0.17 ^e^	32.93 ± 0.49 ^h^	61.64 ± 0.04 ^f^	74.84 ± 0.0018 ^ b ^
G41	3.4 ± 0.20 ^d^	9.29 ± 0.61 ^d^	12.91 ± 0.32 ^f^	31.74 ± 0.40 ^h^	56.86 ± 0.13 ^g^	84.06 ± 0.0016 ^ a ^

Note: The scavenging rate is expressed as the mean ± standard deviation. All experiments were repeated three times, and the IC50 value was the half-inhibitory concentration. VC and BHT were used as positive controls, while the remaining 7 strains served as test samples. Different lowercase letters as a shoulder label in the same row indicate significant differences (*p* < 0.05).

**Table 4:** Scavenging rate of ABTS free radicals by the endophytic fungi from papaya.

Sample Name	ABTS Scavenging Rate, %, at Different Concentrations of Samples					IC50 Value, mg/L
	6.25 mg/L	12.5 mg/L	25 mg/L	50 mg/L	100 mg/L	
VC	45.79 ± 0.72 ^a^	73.17 ± 0.18 ^a^	84.22 ± 0.35 ^a^	98.89 ± 0.1 ^a^	99.6 ± 0.05 ^a^	6.91 ± 0.0012 ^d^
BHT	35.85 ± 0.85 ^b^	47.66 ± 0.53 ^b^	61.78 ± 0.93 ^b^	81.29 ± 0.53 ^b^	93.6 ± 0.20 ^b^	12.86 ± 0.0025 ^d^
Y17	31.72 ± 0.13 ^c^	46.9 ± 0.68 ^b^	60.62 ± 0.46 ^b^	76.8 ± 0.13 ^c^	91.83 ± 0.38 ^c^	14.34 ± 0.0016 ^d^
Y28	17.55 ± 0.17 ^f^	28.04 ± 0.36 ^e^	43.47 ± 0.36 ^c^	55.07 ± 0.35 ^d^	76.25 ± 0.09 ^d^	34.23 ± 0.0043 ^c^
J2	28.34 ± 0.13 ^f^	33.99 ± 0.41 ^f^	40.34 ± 0.79 ^f^	52.75 ± 0.13 ^g^	69.64 ± 0.39 ^g^	36 ± 0.0021 ^c^
G45	20.42 ± 0.43 ^e^	29.55 ± 0.63 ^d^	35.3 ± 0.10 ^e^	49.52 ± 0.68 ^f^	71.66 ± 0.31 ^e^	42.31 ± 0.0012 ^c^
J28	18.91 ± 0.15 ^g^	25.92 ± 0.33 ^g^	31.16 ± 0.26 ^e^	44.68 ± 0.18 ^g^	66.46 ± 0.05 ^g^	54.1 ± 0.0022 ^b^
Y8	11.9 ± 0.22 ^d^	17.35 ± 0.57 ^c^	33.23 ± 0.64 ^d^	45.89 ± 0.66 ^e^	66.92 ± 0.18 ^f^	54.36 ± 0.0032 ^b^
G41	12.71 ± 0.23 ^g^	17.3 ± 0.22 ^g^	25.82 ± 0.18 ^g^	37.32 ± 0.10 ^h^	66.16 ± 0.22 ^g^	64.63 ± 0.0012 ^a^

Note: The scavenging rate is expressed as the mean ± standard deviation. All experiments were repeated three times, and the IC50 value was the half-inhibitory concentration. VC and BHT were used as positive controls, while the remaining 7 strains served as test samples. Different lowercase letters as a shoulder label in the same row indicate significant differences (*p* < 0.05).

**Table 5:** Screening results for the strains with antimicrobial activity.

Fungal Genus	Strain Number	Inhibition Rate (%)				
		*Fusarium oxysporum*	*Neoscytalidium dimidiatum*	*Pestalotiopsis* spp.	*Botryodiplodia theobromae*	*Colletotrichum gloeosporioides* penz.
*Fusarium* spp.	J8	44.16 ± 1.02	-	61.78 ± 1.49	-	-
*Phyllosticta* spp.	J28	-	-	36.44 ± 0.21	48.52 ± 2.49	44.91 ± 4.55
*Penicillium* spp.	Y17	-	53.29 ± 0.98	37.25 ± 1.27	40.62 ± 1.61	54.09 ± 1.45
	Y8	27.51 ± 1.42	-	-	45.86 ± 1.93	
*Aspergillus* spp.	J16	38.27 ± 0.75	68.44 ± 0.47	-	-	58.09 ± 2.80
	J17	-	-	-	46.62 ± 3.13	30.91 ± 2.32
	Y23	23.82 ± 1.56	46.58 ± 1.14	-	-	-
	Y28	-	50.81 ± 1.82	56.11 ± 1.01	48.52 ± 1.95	-
	G5	29.83 ± 2.47	31.63 ± 1.65	-	-	43.00 ± 1.77
	G16	24.62 ± 2.20	-	46.96 ± 1.69	-	53.82 ± 1.14
	G45	-	-	-	45.04 ± 2.05	52.64 ± 2.56
*Phoma* spp.	Y9	41.97 ± 2.27	-	39.68 ± 0.57	-	-
*Candida* spp.	G39	30.87 ± 4.05	-	66.40 ± 1.53	-	-

Note: The inhibition rate is expressed as the mean ± standard deviation; “-” means that there was no fungal activity.

## Data Availability

Data are contained within the article and Supplementary Materials.

## Supplementary Materials

The following supporting information can be downloaded at: https://www.mdpi.com/article/10.3390/jof10080550/s1, Figure S1: PCR amplified fragments of rDNA ITS regions; Table S1: NCBI login number of 131 strains isolated from papaya. Appendix S1: Endophytic fungi Y17 rDNA ITS sequence.
