# Supplementary material for: Research on the Isolation of Endophytic Fungi from Papaya and the Prevention of Colletotrichum gloeosporioides
Source: J Fungi (Basel). 2024 Aug 5;10(8):550. doi: 10.3390/jof10080550 (PMC11355454; doi:10.3390/jof10080550)
Supplement: Supplementary file 1 [file jof-10-00550-s001.zip › jof-2965835-supplementary.pdf]

Table S1. NCBI login number of 131 strains isolated from papaya

Number	Strain	NCBI-ID	Number	Strain	NCBI-ID	Number	Strain	NCBI-ID
1	G52	MT729829	46	G25	MT729874	91	Y38	MT729919
2	G50	MT729830	47	G16	MT729875	92	Y28	MT729920
3	G45	MT729831	48	G2	MT729876	93	Y27	MT729921
4	G53	MT729832	49	G1	MT729877	94	Y4	MT729922
5	G32	MT729833	50	G19	MT729878	95	Y1	MT729923
6	G30	MT729834	51	G55	MT729879	96	Y32	MT729924
7	G29	MT729835	52	G48	MT729880	97	Y42	MT729925
8	G42	MT729836	53	G47	MT729881	98	Y25	MT729926
9	G41	MT729837	54	G43	MT729882	99	Y7	MT729927
10	G39	MT729838	55	G34	MT729883	100	Y5	MT729928
11	G33	MT729839	56	J24	MT729884	101	Y30	MT729929
12	G44	MT729840	57	J30	MT729885	102	Y37	MT729930
13	G20	MT729841	58	J12	MT729886	103	Y24	MT729931

14	G6	MT729842	59	J15	MT729887	104	Y3	MT729932
15	G15	MT729843	60	J3	MT729888	105	Y9	MT729933
16	G14	MT729844	61	J10	MT729889	106	Y6	MT729934
17	G12	MT729845	62	J11	MT729890	107	Y2	MT729935
18	G10	MT729846	63	J5	MT729891	108	Y11	MT729936
19	G8	MT729847	64	J26	MT729892	109	Y12	MT729937
20	G23	MT729848	65	J31	MT729893	110	Y13	MT729938
21	G18	MT729849	66	J28	MT729894	111	Y14	MT729939
22	G31	MT729850	67	J1	MT729895	112	Y18	MT729940
23	G3	MT729851	68	J4	MT729896	113	Y19	MT729941
24	G49	MT729852	69	J25	MT729897	114	Y22	MT729942
25	G24	MT729853	70	J18	MT729898	115	Y23	MT729943
26	G46	MT729854	71	J7	MT729899	116	Y29	MT729944
27	G40	MT729855	72	J9	MT729900	117	Y39	MT729945
28	G36	MT729856	73	J19	MT729901	118	Y44	MT729946

29	G35	MT729857	74	J23	MT729902	119	Y43	MT729947
30	G27	MT729858	75	J6	MT729903	120	Y34	MT729948
31	G22	MT729859	76	J8	MT729904	121	Y33	MT729949
32	G21	MT729860	77	J14	MT729905	122	Y31	MT729950
33	G17	MT729861	78	J16	MT729906	123	Y21	MT729951
34	G13	MT729862	79	J17	MT729907	124	Y20	MT729952
35	G11	MT729863	80	J21	MT729908	125	Y17	MT729953
36	G9	MT729864	81	J22	MT729909	126	Y16	MT729954
37	G7	MT729865	82	J29	MT729910	127	Y15	MT729955
38	G5	MT729866	83	J13	MT729911	128	Y10	MT729956
39	G4	MT729867	84	J2	MT729912	129	Y26	MT729957
40	G54	MT729868	85	J27	MT729913	130	Y36	MT729958
41	G51	MT729869	86	J20	MT729914	131	Y8	MT729959
42	G38	MT729870	87	Y35	MT729915			
43	G37	MT729871	88	Y45	MT729916			

44	G28	MT729872	89	Y41	MT729917			
45	G26	MT729873	90	Y40	MT729918			

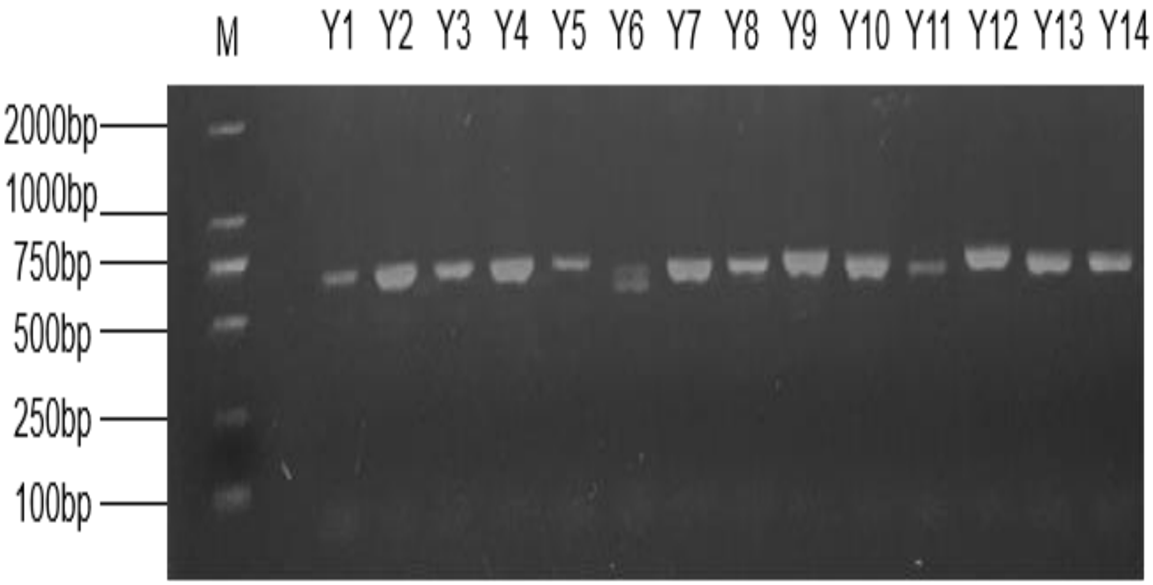

Figure S1. PCR amplified fragments of rDNA ITS regions

Appendix S1. Endophytic fungi Y17 rDNA ITS sequence

>Y17.ITS

TAACGAAGTGAGGACTCTGGGTCACCTCCCACCCGTGTTTATCGTACCTTGTTGCTTCGGCGAGCCCGCCTCACGGCCGC

CGGGGGGCATCCGCCCCCGGGCCCGCGCTCGCCGAAGACACCATTGAACTCTGTCTGAAGATTGCAGTCTGAGTGATTAA

CTAAATCAGTTAAAACTTTCAACAACGGATCTCTTGGTTCCGGCATCGATGAAGAACGCAGCGAAATGCGATAAGTAATG

TGAATTGCAGAATTCAGTGAATCATCGAGTCTTTGAACGCACATTGCGCCCCCTGGTATTCCGGGGGGCATGCCTGTCCG

AGCGTCATTGCTGCCCTCAAGCACGGCTTGTGTGTTGGGCCCCGCCCCCCGGTTCCGGGGGGCGGACCCGAAAGGCAGCG

GCGGCACCGCGTCCGGTCCTCGAGCGTATGGGGCTTCGTCACCCGCTCTGTAGGCCCGGCCGGCGCCCGCCGGCGACCCC

AATCAATCTATCCAGGTTGACCTCGGATCAGGTAGGGATACCCGCTGAACTTAAGCATATCAATAAGCGGAGGA
